# High Temperature Tensile Fracture Behavior of Copper-Containing Austenitic Antibacterial Stainless Steel

**DOI:** 10.3390/ma15041297

**Published:** 2022-02-10

**Authors:** Jiali Qian, Haijuan Wang, Jing Li, Rongjun Xu

**Affiliations:** 1 State Key Laboratory of Advanced Metallurgy, University of Science and Technology Beijing (USTB), Beijing 100083, China; qjl18395589035@163.com; 2 School of Metallurgical and Ecological Engineering, University of Science and Technology Beijing (USTB), Beijing 100083, China; 3 Guangdong Guangqing Metal Technology Co., Ltd., Yangjiang 529500, China; xurongjun_gqmt@163.com

**Keywords:** 304 Cu-containing stainless steel, thermoplastic, high temperature tensile, ferrite

## Abstract

The mechanical properties and deformation microstructure of cast 304 Cu-containing austenitic stainless steel at 10^−3^/s strain rate in the range of 700~1200 °C were studied by Gleeble thermal simulator, metallographic microscope and scanning electron microscope. The results showed that the thermoplasticity of 304 Cu-containing austenitic stainless steel was higher than 60% when the temperature was higher than 1000 °C, and the tensile strength as a whole decreased with the increase in temperature. During the tensile process, the morphology and content of ferrite in the test steel were the main factors affecting the high-temperature thermoplastic of the billet. The inclusions near the fracture and the existence of ferrite at the grain boundary greatly affected the formation of microcracks and holes and the fracture.

## 1. Introduction

Due to its excellent corrosion resistance and broad-spectrum antibacterial properties, Copper (Cu)-containing austenitic antibacterial stainless steel has been used extensively in many fields, such as kitchen appliances, food processing, medical health, and public facilities [1,2,3]. However, Cu-rich phase with a low-melting point are formed in Cu-containing austenite antibacterial stainless steel due to the selective enrichment of Cu elements, which is prone to surface cracks during the continuous casting production process [4,5,6]. These cracks seriously affect the subsequent processing of the slab and, furtherly, reduce the quality of the finished product.

The crack sensitivity of the billet is directly related to the high-temperature tensile strength and thermoplasticity of the steel [7,8,9,10]. Yang [11] found that the 0Cr18Ni9 antibacterial stainless steel containing 3.71 wt.% Cu obtained good thermoplasticity at 1050~1200 °C, and excellent section shrinkage rate, which obviously avoided the generation of cracks in the subsequent single-pass rolling process. Dong [12] proved that the tensile strength of steel should be guaranteed to be higher than 25 MPa, which was to avoid cracks caused by non-uniform deformation of 304 stainless steel at high temperatures. Liu [13] found the tensile test temperature range was small as the shrinkage rate was higher than 60% of the J4 stainless steel, which indicated that the steel had a strong crack sensitivity and poor tensile crack resistance. Thus, for the J4 stainless steel, the temperature of the secondary cooling zone should be controlled within the plastic temperature range to reduce cracks during straightening. The thermoplasticity of materials and the generation of cracks are affected by many factors [14,15,16], for instance, the low-melting-point eutectic phase formed at grain boundary due to the segregation of impurity elements such as S and P, the second phase particles precipitated during thermal deformation, the external deformation conditions exceeding the deformation capacity of the material, etc. For example, Yang [17] compared the micro-structures of the brittle fracture zone and the plastic deformation zone of titanium-containing microalloyed steel, and found that the intergranular ferrite greatly deteriorated the steel plasticity and promoted the occurrence of the intergranular brittle fracture.

However, lots of research into Cu-bearing austenitic antibacterial stainless steel has been focused on the effect of Cu content and heat treatment process on the mechanical properties, and the relationship between the microstructures and the tensile fracture behavior was rarely reported. Therefore, in this paper, 304 Cu-containing antibacterial stainless steel casting billet was used as the research object, and the high-temperature thermoplasticity of as-cast 304-Cu stainless steel at different temperatures was measured by high-temperature tensile test. The morphology and microstructure of the fracture were observed to study the thermoplastic interval and tensile cracks generation mechanism of 304-Cu, which could provide a reference for the reasonable selection of continuous casting process parameters. Furtherly, the corresponding relationship of tensile behavior and microstructural evolution at high temperatures was clarified.

## 2. Experimental Procedure

**Figure 1:**
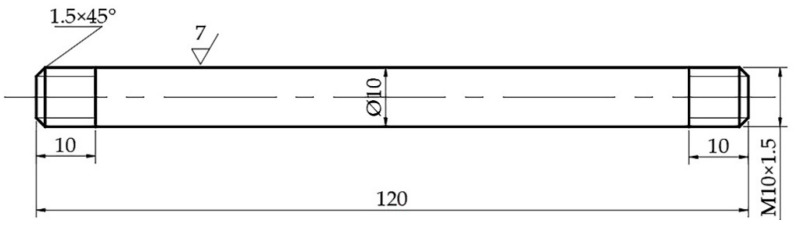
Sample processing drawing.

The experimental steel, 304 Cu-containing austenitic antibacterial stainless steel, was an industrial production. The production process was as follows: electric arc furnace (EAF)—argon oxygen decarburization (AOD) smelting—ladle furnace (LF)—continuous casting. The chemical composition of the steel is listed in Table 1. Samples were taken from the cast billet in this study. In order to avoid the influence of center segregation on the experiment, this experiment avoided the central area of the slab thickness, and samples were taken from 1/4 of the slab thickness direction, and the long axis of the sample was parallel to the width direction of the slab. To investigate the influence of temperature on the mechanical properties of 304-Cu, samples were machined into a round bar sample of Φ10 mm × 120 mm (M10 threads with a length of 10 mm at both ends) at high temperatures, as shown in Figure 1.

**Figure 2:**
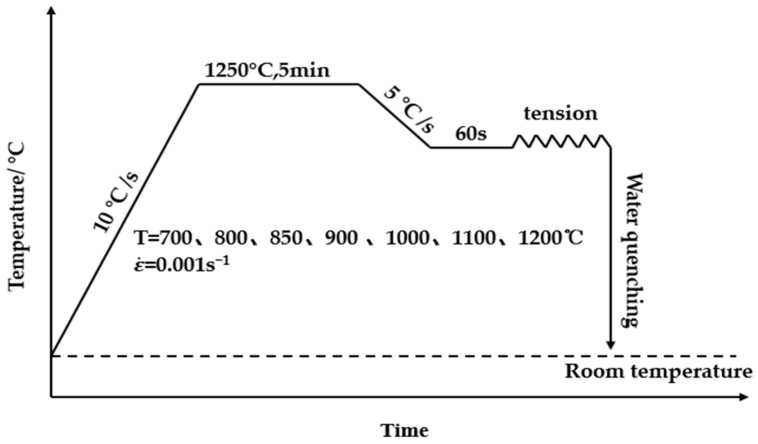
High temperature drawing process curve.

The high-temperature tensile tests were carried out using the Gleeble-1500 thermal simulator under a pure argon protective atmosphere, and the specific test heating system is shown in Figure 2. Samples were heated to 1250 °C at a heating rate of 10 °C/s, and kept for 5 min to uniformly heat the temperature. In order to eliminate the temperature gradient, samples were kept at the target tensile temperature for 60 s firstly and then the tensile tests at a strain rate of 10^−3^/s were performed. In order to ensure the morphology and metallographic characteristics of the fracture at high temperature were retained, the tensile tests samples were water-cooled immediately after being broken. Three tensile tests were performed at each temperature.

After the tensile tests, the fracture diameter of the tensile sample was measured with a spiral micrometer, the section of the sample at different temperatures was calculated and the high-temperature plastic curve of the test steel was drawn. After the fracture was cleaned by an ultrasonic cleaning instrument, the tensile fracture morphology was observed by scanning electron microscope (SEM, FEI quanta 250; FEI Corporation, Hillsboro, OR, USA), and the fracture mechanism was analyzed. The tensile sample was cut along the axial direction and the area near the fracture was ground and polished. The metallographic structure of the fracture area was revealed via etching using a mixed solution of 10% sulfuric acid aqueous solution (100 mL) and potassium permanganate (2 g), and then observed using the metallographic microscope (OM, DM4M, Leica, Wetzlar, Germany).

## 3. Results and Discussion

### 3.1. Thermoplastic Curve

The section shrinkage rate is an important performance index to measure the plastic deformation ability of a material, which reflects the plastic deformation ability of the cast slab at high temperature. The tensile strength reflects the maximum stress value that the material can bear and the ability of the cast slab to resist the generation and propagation of cracks. According to previous studies [18,19,20], when the section shrinkage rate is less than 60%, the crack sensitivity of the cast slab increases with its decrease. Therefore, the critical value of 60% was used in this study for dividing the high-plastic zone and the low-plastic zone.

**Figure 3:**
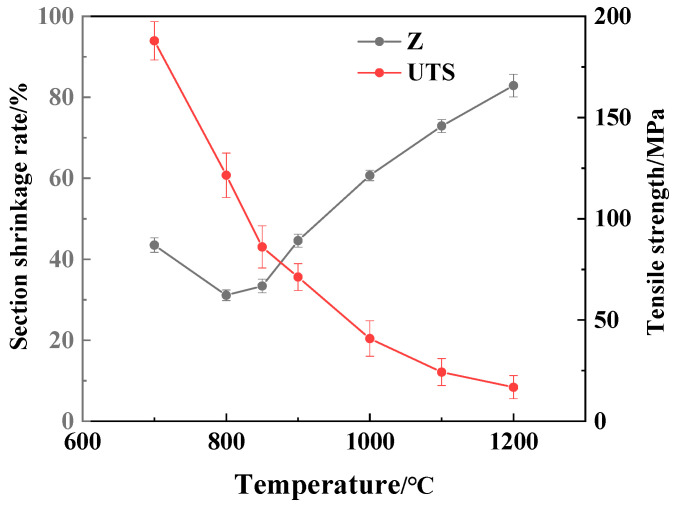
Section shrinkage rate and tensile strength at different temperature.

Figure 3 shows the curve of the section shrinkage rate (Z) and the tensile strength (UTS) of 304-Cu stainless steel casting billets with temperature. It can be seen from Figure 3 that in the temperature range of 1000~1200 °C, the section shrinkage rate of 304-Cu increased gradually, which were higher than 60%. In the temperature range of 700~900 °C, the plasticity of the cast slab decreased significantly. As the temperature raised, the section shrinkage rate decreased firstly and then increased, with a minimum value of 31.1%, which was the third brittle zone. When the strain rate was less than 1 × 10^−2^/s, the third brittle zone was very likely to appear in the steel. In this temperature range, the high-temperature thermoplastic of the steel was greatly related to the cracks in the continuous casting slab. Therefore, an appropriate secondary cooling system should be adopted in production to reduce the generation of slab surface microcracks. In the process of continuous casting bending and straightening, the slab surface temperature should be controlled above 1000 °C to avoid the formation of third brittle zone.

It can also be seen in Figure 3 that the tensile strength decreased with increasing temperature. This result can be explained that the kinetic energy of atoms is reduced with the decreasing temperature, thus the bonding force between atoms increases, and the critical shear stress increases. The decrease in temperature will slow down the recovery and recrystallization process of the material, reducing its softening effect on the material. When the temperature was higher than 1100 °C, the tensile strength of the cast slab was smaller than 25 MPa, which indicated that the cast slab was liable to exceed its high-temperature strength limit under high temperatures, resulting in non-uniform plastic deformation and cracking defects.

### 3.2. Stress-Strain Curve

**Figure 4:**
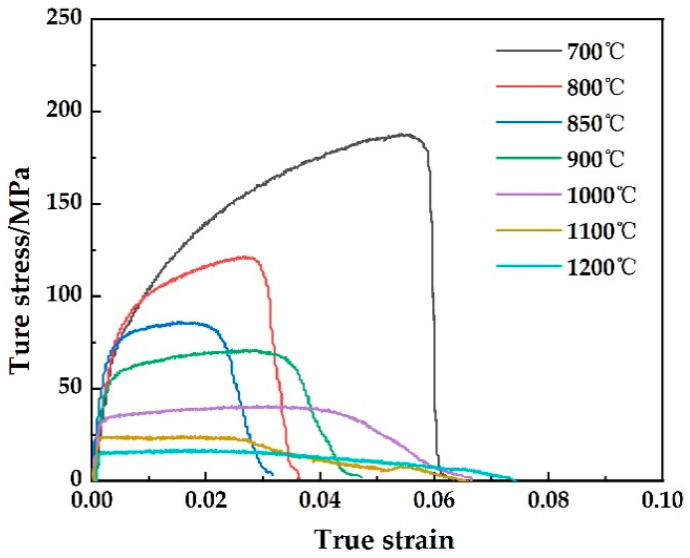
Stress-strain of 304-Cu at different temperatures.

Figure 4 shows the uniaxial tensile stress-strain curve of 304-Cu stainless steel at different temperatures. When the test temperature increases, the peak stress of the material decreases. Temperature greatly affected the comprehensive effects of deformation strengthening, recovery and recrystallization, causing a particularly prominent impact on the high-temperature mechanical properties of materials. Moreover, the deformation mechanism changed at various temperatures, leading to the completely different tensile behavior of samples. When the temperature was lower than 1000 °C, the curve showed dynamic recovery type, and the stress began to decrease sharply after increasing from zero to a certain value. When the temperature was higher than 1000 °C, the curve showed dynamic recrystallization type. After the stress increased from zero to a certain value, it began to decrease slowly with the continuous increase in strain. The elongation of the specimen increased greatly when the temperature was higher than 1000 °C. By comparing the stress-strain curve with Figure 3, it can be seen that the variation trend of section shrinkage was consistent with that of elongation, indicating that the high temperature thermoplasticity of steel above 1000 °C was excellent.

### 3.3. Fracture Morphologies

**Figure 5:**
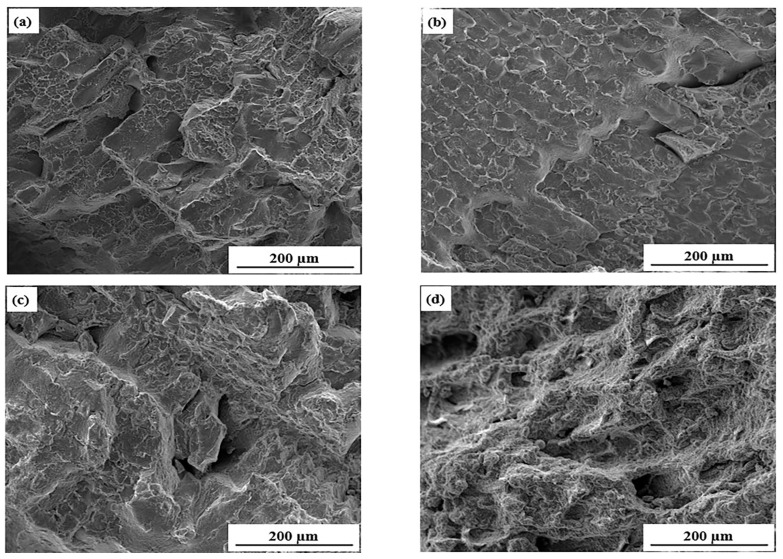

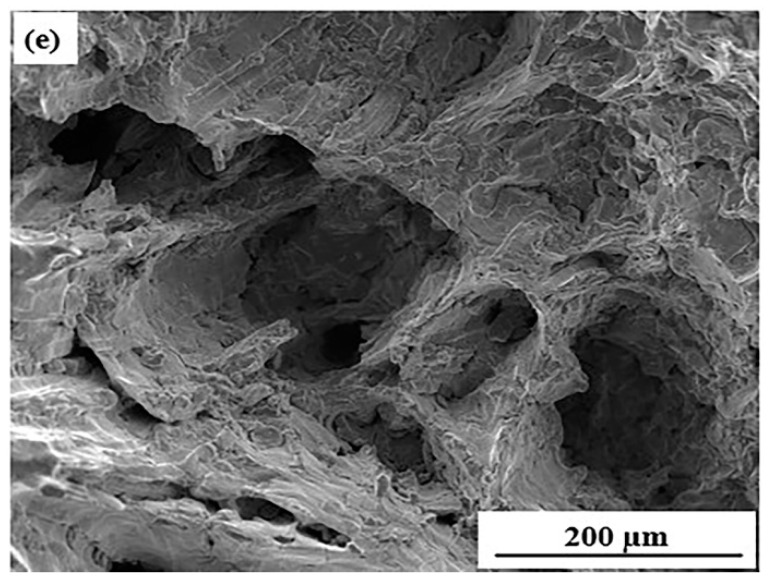
Fracture morphologies test sample at 500 times magnification: (**a**) 700 °C, (**b**) 800 °C, (**c**) 900 °C, (**d**) 1000 °C, (**e**) 1100 °C.

In order to investigate the fracture mechanism of the samples at different temperatures, the fracture micromorphology of the test steel at different temperatures was observed, as shown in Figure 5.

As shown in Figure 5a,c, the fracture morphologies of the samples at 700 and 900 °C were mainly rock sugar-like, with similar wedge-shaped creep cracks. The grain interface was flat, and the fracture was short and not continuous in river shape. Therefore, the fracture at these temperatures was a quasi-cleavage fracture. Quasi-cleavage fracture is a kind of transgranular fracture between a ductile fracture and a brittle fracture; thus, it presented a mid performance between ductile fracture and brittle fracture. Figure 5b shows the fracture of the sample at 800 °C. It can be seen that the surface of the fracture was flat, which was similar to the shape of neatly arranged crystal grains. The plastic deformation around the fracture was not obvious at 800 °C, though there were obvious intergranular cracks between grains, which was a typical intergranular brittle fracture.

Figure 5d shows the fracture of the sample at 1000 °C. The fracture mode of the sample was microporous aggregation fracture. Compared to Figure 5a–c, much more small and uneven small dimples could be observed on the fracture, which indicated that a better thermoplasticity of the sample occurred with the increase in temperature. There were large and deep dimples with irregularly distributed in Figure 5e. The increase in dimples indicated that the relative distance between the holes was far away at 1100 °C, meaning that the defects have experienced sufficient growth after nucleation and the crack resistance was strong. At this time, the fracture had a tearing ridge, and the sample had good plasticity.

**Figure 6:**
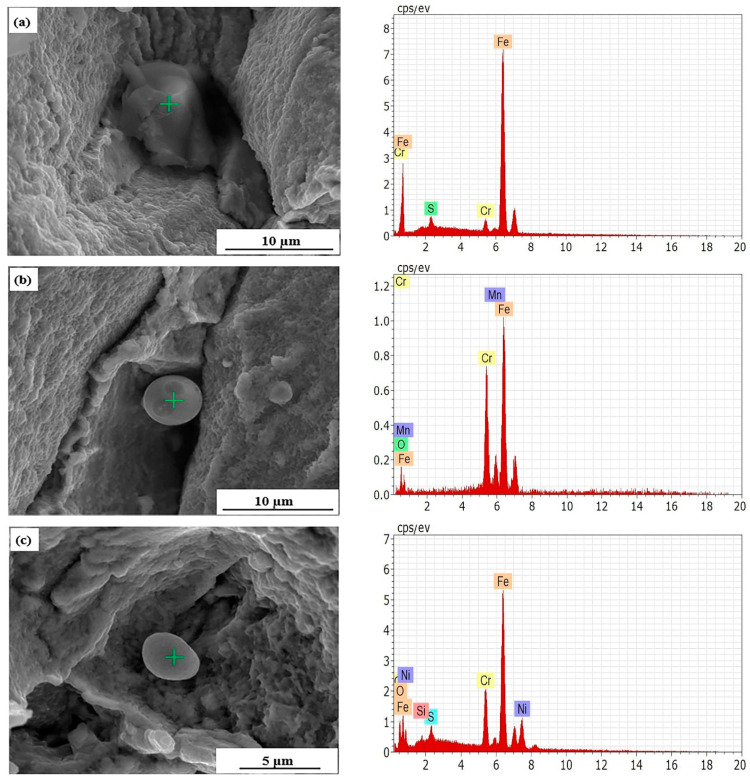
EDS analysis of particles at the tensile fracture: (**a**) 800 °C, 9500×, (**b**) 900 °C, 10,000×, (**c**) 1000 °C, 15,000×.

Figure 6 shows the morphology and composition of particles near the fracture. It can be seen that the particles were mainly composed of sulfide and oxide. After tensile load, the grains slip greatly and produce dislocation accumulation under the action of stress, resulting in many micropores in local areas such as necking. At the same time, when the grain boundary stress exceeds the strength of the inclusion, the inclusion will break and form microcracks. These micropores and microcracks grew, deformed and connected with each other under the action of external stress, finally causing the whole material to break under the action of tension [20,21]. The fracture of the sample at a high temperature was mainly related to the number and distribution of voids formed by the further growth of micropores, and the generation of voids was closely related to the grain boundary properties of the matrix. If the matrix grain boundary is greatly hindered and difficult to move during the sliding process, and the recrystallization process is slow, the grain boundary sliding will be carried out in the form of deformation, resulting in cracks at the intersection of the grain boundary or holes along the grain boundary.

### 3.4. Fracture Microstructure Analysis

**Figure 7:**
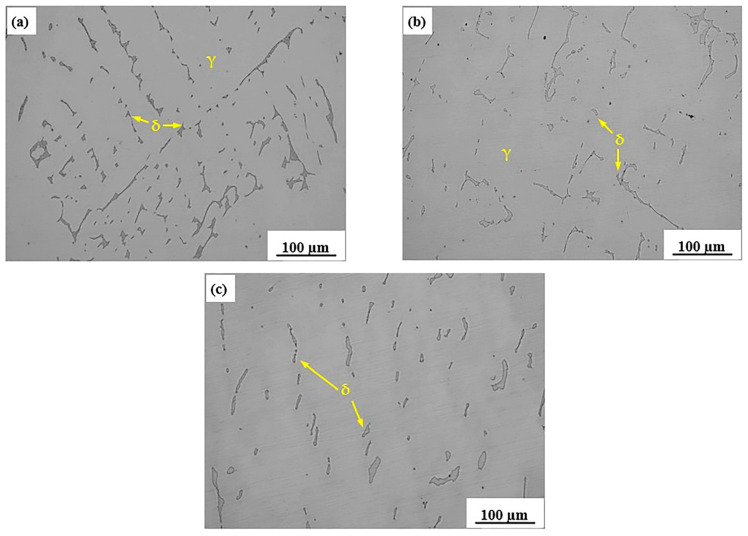
Metallographic diagram of 304-Cu stainless steel at 200 times magnification: (**a**) 800 °C, (**b**) 900 °C, (**c**) 1100 °C.

Figure 7 shows the metallographic structure of the sample at different temperatures. It can be seen that the shape of ferrite changed gradually at different temperatures. When the temperature was 1100 °C, the ferrite was granular and distributed uniformly in the austenite matrix. When the temperature decreased to 900 °C, the ferrite aggregated with each other, and the granular ferrite connected into strips. When the temperature furtherly decreased to 800 °C, the strip ferrite had been connected into a network structure. Previous studies have indicated [22,23,24,25] that the ferrite phase played a crucial role on the thermoplasticity of steel, and the shape and content of ferrite produced during the heat preservation and heating process greatly affected the generation of cracks. The high temperature plasticity of austenitic stainless steel could be improved obviously when the content of ferrite was low and distributed evenly in the matrix in a granular form. However, when the ferrite distributed in a network structure, the thermoplasticity of the steel decreased significantly.

**Figure 8:**
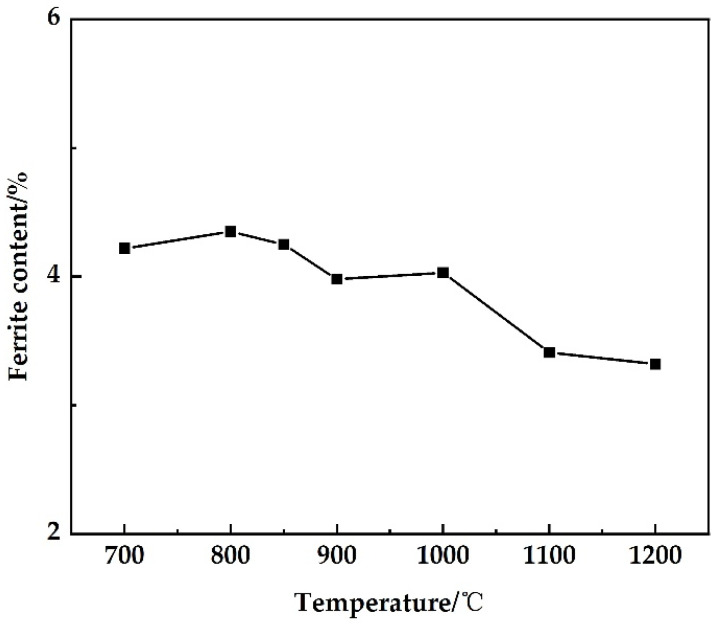
The relationship between ferrite content and temperature in 304 stainless steel.

The ferrite content in the steel at each stretching temperature was counted by Image-Pro Plus 6.0 (IPP 6.0, Media Cybernetics, Inc., Rockville, MD, USA) software with 15 metallographic photos. The statistical results are shown in Figure 8. It can be seen from Figure 8 that as the temperature increased, the ferrite content showed a downward trend as a whole. In 304 and high-N low-Ni austenitic stainless steel-containing Cr 19 wt.%, it was found that 2% ferrite would lead to a decrease in thermal plasticity [26]. The higher the ferrite content, the more significant the decrease in thermal plasticity [24]. Therefore, based on the section shrinkage rate curve, it can be known that the shape and content of ferrite are the main factors affecting the high-temperature mechanical properties of 304-Cu.

**Figure 9:**
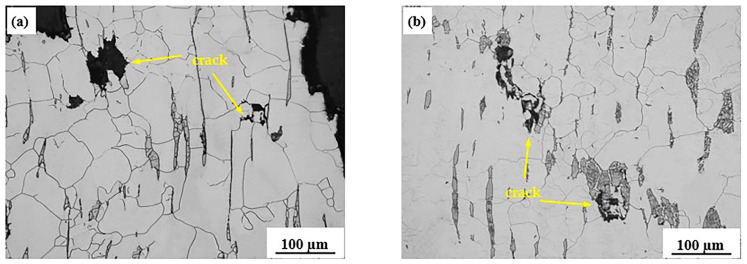

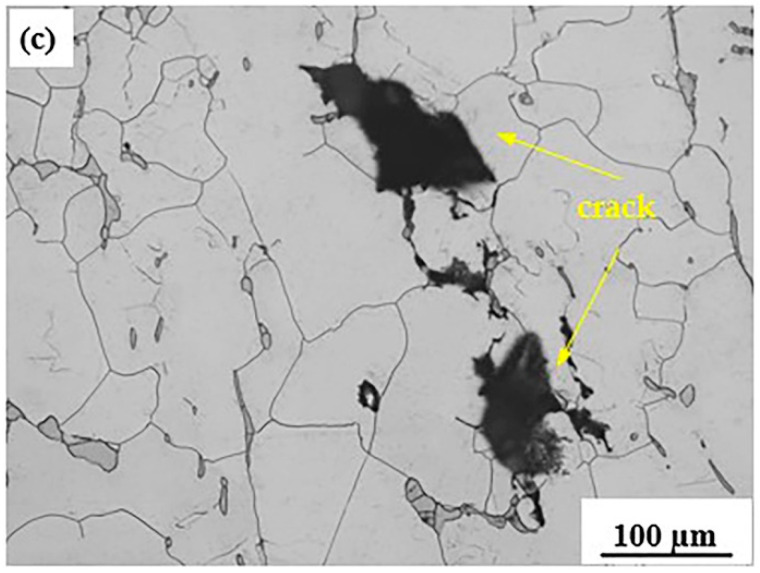
Metallographic diagram of fracture zone at 200 times magnification: (**a**) 1000 °C, (**b**) 1100 °C, (**c**) 1200 °C.

The tensile sample was cut along the tensile direction, and the internal residual cracks and microstructure were observed, as shown in Figure 9. It was found that the cracks are mainly located at the ferrite part of the austenite grain boundary and the intersection of the three crystals. This result can be explained in that there are great differences in deformation capacity between ferrite and austenite, as the strength of ferrite is only 1/4 of that of austenite. When ferrite is distributed on the austenite grain boundary, the deformation under the stress of the sample during tensile deformation preferentially occurs on the ferrite generated on the austenite grain boundary. This deformation preferentially occurs on the matrix grain boundary which will make the ferrite vulnerable to stress concentration, so the ferrite is susceptible to be in the area with stress concentration during the process of tensile deformation, thus resulting in fractures and cracks. Under the influence of stress, the material will eventually fracture along these cracks and propagation areas [18,27]. In addition, during the process the deformation, the intersecting areas of grain boundaries were prone to stress concentration due to the grain boundary slipping, which also led to the generation of cracks.

## 4. Conclusions

In this paper, the high-temperature tensile fracture mechanism of 304 copper-containing austenitic antibacterial stainless steel was investigated. The main conclusions of the current work are as follows:

(1) The tensile strength of as-cast 304-Cu austenitic stainless steel gradually decreased with the increase in temperature. The temperature range of the third brittle zone of 304-Cu was 700~900 °C. At 1000~1200 °C, the Section shrinkage rate was more than 60%, and the plasticity was excellent. In the bending section and correction of continuous casting production, the surface temperature of the cast slab should be controlled toreach higher than 1000 °C to reduce the occurrence of slab surface defects.
(2) Inclusions on the fractures of ductile fracture and brittle fracture were observed in tensile samples, which indicated that the inclusions were the source of microcracks in the fracture process. When the stress exceeded the strength of the inclusions, holes were generated in the matrix.
(3) The high temperature thermoplasticity of 304-Cu was greatly affected by the morphology and content of ferrite, and the cracks were mainly formed at the grain boundary of ferrite and the intersection of the grain boundary. The reticulated ferrite obviously reduced the high-temperature thermoplasticity of steel. As the temperature increased, the ferrite content gradually decreased and the morphologies of ferrite were transformed from network to granular, which contributed to the improvement of high-temperature thermoplasticity of 304-Cu significantly.

## Figures and Tables

**Table 1:** Chemical compositions of 304-Cu antibacterial stainless steel (wt.%).

C	Si	Mn	P	S	Cr	Ni	Cu	N
0.024	0.48	0.68	0.025	0.03	17.46	8.00	3.80	0.030

## Data Availability

Not applicable.

## References

[1] Yang K., Lu M.Q. (2007). Antibacterial properties of an austenitic antibacterial stainless steel and its security for human boby. Mater. Sci. Technol..

[2] Li N., Yang W., Liu Y., Hui X.U., Yang K. (2008). Antibacterial mechanism of copper-bearing antibacterial stainless steel against *E. coli*. Mater. Sci. Technol..

[3] Hong I.T., Koo C.H. (2005). Antibacterial properties, Corrosion resistance and Mechanical properties of Cu-Modified SUS 304 stainless steel. Mater. Sci. Eng..

[4] Di H.S., Cui G.Z., Wang G.D., Liu X.H. (2003). Hot ductility of 304HC stainless steel and The model of resistance to deformation. Acta Metall. Sin..

[5] Hou G.Q., Zhu H.L. (2013). Hot ductility and Microstructure in slab shell of low Ni austenitic stainless steel. Adv. Mater. Res..

[6] Luo F.F., Tang Z.H., Xiao S.F., Xiang Y.L. (2019). Study on properties of copper-containing austenitic antibacterial stainless steel. Mater. Technol..

[7] Mintz B., Yue S., Joans J.J. (1991). Hot ductility of steels and Its relationship to the problem of transverse craking during continous casting. Int. Mater. Rev..

[8] Suzuki H.G., Nishimura S., Yamaguchi S. (1979). Characteristics of embrittlement in steels above 600 °C. Tetsu-Hagane.

[9] Royzman S.E. (1996). Shrinking stress in a solidfying continous casting slab. Steel Technol. Intern..

[10] Won Y.M., Kim K.H., Yeo T.J. (2007). Effect of cooling rate on ZST, LIT and ZDT of carbon steels near melting point. ISIJ Int..

[11] Yang K., Dong J.S., Chen S.H., Lv M.Q. (2006). The craftwork performance and resistance to corrosion of the Cu-containing antibacterial stainless steels. Chin. J. Mater. Res..

[12] Dong F., Qi J.M., Xin R.F. (2014). Study on high temperature mechanical properties of 304 stainless steel. Hot Work. Technol..

[13] Liu P. (2009). Study on J4 stainless steel continuous casting flux. Master Degree Dissertation.

[14] Yazdani M., Abbasi S.M., Momeni A., Taheri A.K. (2011). Hot ductility of a Fe-Ni-Co alloy in cast and wrought conditions. Mater. Des..

[15] Pei H.X., Zhang H.L., Wang L.X., Li S.L., Li D.Z., Wang X.T. (2014). Tensile behaviour of 316LN stainless steel at elevated temperatures. Mater. High Temp..

[16] Hu G.D., Wang P., Li D.Z., Li Y.Y. (2019). Effects of nitrogen on precipitation and tensile behaviors of 25Cr-20Ni austenitic stainless steels at elevated temperatures. Mater. Sci. Eng. A.

[17] Yang X.G., Zhang L.F., Ren Y., Li S.S., Li M. (2016). Hot ductility and fracture mechanism of a Ti-bearing microalloyed steel. Chin. J. Eng..

[18] Li Y.F., Zeng X.G. (2019). Dynamic tensile behavior and constitutive modeling of TC21 titanium alloy. Wuhan Univ. Technol.-Mater. Sci. Ed..

[19] Suzuki H.G., Nishimura S., Imamura J. (2010). Hot ductility in steels in the temperature range between 900 and 600 °C. Tetsu-Hagane.

[20] Shin A.D., Barter M.C., Kenny J.M. (2000). Effects of grain size on the properties of a low nickel austenitic stainless steel. J. Mater. Sci..

[21] Sakai T., Jonas J.J. (1984). Dynamic recrystallization mechanical and microstructural considerations. Acta Met. Mater..

[22] Sheng J., Li J.C., La P.Q., Wei F., Song Y., Wang K.L. (2019). Investigating the tensile properties of micro-nanostructured 304 stainless steel with SEM and in-situ tension. Sci. Adv. Mater..

[23] Ma F.J., Wen G.H., Wang W.L. (2013). Effect of cooling rates on the second-phase precipitation and proeutectoid phase transformation of a NbTi microalloyed steel slab. Steel Res. Int..

[24] Czerwinski F., Cho J.Y., Brodtka A., Zielinska-Lipiec A., Sunwoo J.H., Szpunar J.A. (1999). The edge-cracking of AISI 304 stainless stell during hot-rolling. J. Mater. Sci..

[25] Yoo J., Choi W.M., Sohn S.S., Lee B.J. (2019). Effects of V or Cu addition on high-temperature tensile properties of high-Ni-containing austenitic cast steels used for high-performance turbo-charger housings. Met. Mater. Int..

[26] Tarboton J.N., Matthews L.M., Sutcliffe A., Frost C.M., Wessels J.P. (1999). The hot workability of Cromanite, a high Nitrogen austenitic stainless steel. Mater. Sci. Forum.

[27] Zhang L., Yang X., Li S., Li M., Ma W. (2014). Control of transverse corner cracks on low-carbon steel slabs. JOM J. Miner. Met. Mater. Soc..

